# 1*H*-Benzimidazol-2-ylmethyl phenyl ether

**DOI:** 10.1107/S1600536809007922

**Published:** 2009-03-11

**Authors:** Noor Doha Hassan, Hairul Anuar Tajuddin, Zanariah Abdullah, Seik Weng Ng

**Affiliations:** a Department of Chemistry, University of Malaya, 50603 Kuala Lumpur, Malaysia

## Abstract

There are two mol­ecules in the asymmetric unit of the title compound, C_14_H_10_N_2_O: the dihedral angles between their aromatic ring planes are 47.4 (4) and 46.8 (3)°. In the crystal structure, mol­ecules are linked by N—H⋯N hydrogen bonds from the secondary nitro­gen N—H donor to the tertiary N-atom acceptor of a symmetry-related neighbour, resulting in hydrogen-bonded chains. The two independent chains both propagate in [100].

## Related literature

For related phen­oxy-substituted *N*-heterocycles, see: Abdullah & Ng (2008 ▶); Hassan *et al.* (2008 ▶); Idris *et al.* (2009 ▶); Shah Bakhtiar *et al.* (2009 ▶).
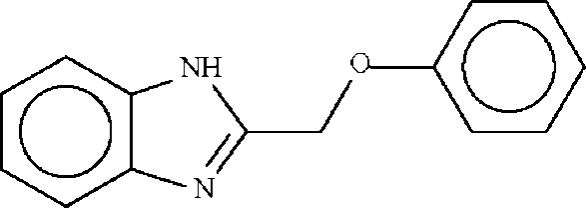


## Experimental

### 

#### Crystal data

C_14_H_12_N_2_O
*M* *_r_* = 224.26
Orthorhombic, 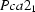

*a* = 10.0299 (5) Å
*b* = 8.5391 (4) Å
*c* = 27.000 (1) Å
*V* = 2312.5 (2) Å^3^
*Z* = 8
Mo *K*α radiation
μ = 0.08 mm^−1^
*T* = 120 K
0.40 × 0.10 × 0.03 mm

#### Data collection

Bruker SMART APEX CCD diffractometer
Absorption correction: none
15022 measured reflections
2699 independent reflections
1783 reflections with *I* > 2σ(*I*)
*R*_int_ = 0.091

#### Refinement

*R*[*F* ^2^ > 2σ(*F* ^2^)] = 0.046
*wR*(*F* ^2^) = 0.111
*S* = 1.00
2699 reflections
313 parameters
3 restraints
H atoms treated by a mixture of independent and constrained refinement
Δρ_max_ = 0.19 e Å^−3^
Δρ_min_ = −0.23 e Å^−3^

### 

Data collection: *APEX2* (Bruker, 2007 ▶); cell refinement: *SAINT* (Bruker, 2007 ▶); data reduction: *SAINT*; program(s) used to solve structure: *SHELXS97* (Sheldrick, 2008 ▶); program(s) used to refine structure: *SHELXL97* (Sheldrick, 2008 ▶); molecular graphics: *X-SEED* (Barbour, 2001 ▶); software used to prepare material for publication: *publCIF* (Westrip, 2009 ▶).

## Supplementary Material

Crystal structure: contains datablocks global, I. DOI: 10.1107/S1600536809007922/hb2923sup1.cif

Structure factors: contains datablocks I. DOI: 10.1107/S1600536809007922/hb2923Isup2.hkl

Additional supplementary materials:  crystallographic information; 3D view; checkCIF report

## Figures and Tables

**Table 1:** Hydrogen-bond geometry (Å, °)

*D*—H⋯*A*	*D*—H	H⋯*A*	*D*⋯*A*	*D*—H⋯*A*
N1—H1⋯N2^i^	0.88 (1)	2.03 (2)	2.879 (7)	163 (6)
N3—H3⋯N4^ii^	0.88 (1)	1.97 (2)	2.845 (8)	172 (5)

Symmetry codes: (i) 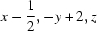; (ii) 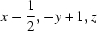.

**Fig. 1.**
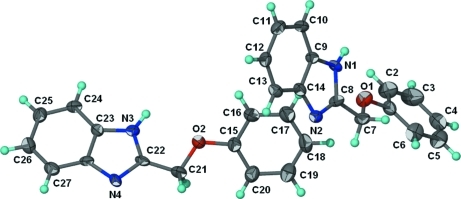
The molecular structure of (I) with displacement ellipsoids shown at the 70% probability level. Hydrogen atoms are drawn as spheres of arbitrary radius.

## supplementary crystallographic information

### Experimental

Phenol (1.88 g, 20 mmol) was mixed with sodium hydroxide (0.08 g, 20 mmol) in several drops of water. The water was then evaporated. The paste was heated with 2-(chloromethyl)benzimidazole 3.30 g, 20 mmol) at 423–433 K for 6 h. The product was dissolved in water and the solution extracted with chloroform. The chloroform phase was dried over sodium sulfate; the evaporation of the solvent a brown product; this was purified by column chromatography with an ethyl acetate/hexane mixture. Crystals were grown from this solvent system gave well shaped colorless crystals along with some unidentified brown material.

### Refinement

Anomalous dispersion was negligible and Friedel pairs were merged before refinement.

The C-bound H-atoms were placed in calculated positions (C—H 0.95–98 Å) and refined as riding with *U*(H) = 1.2*U*_eq_(C). The N-bound H atoms were located in a difference map, and were refined with a restraint of N–H 0.88±0.01 Å; their U_iso_ values were freely refined.

### Crystal data

**Table:**

C_14_H_12_N_2_O	*F*(000) = 944
*M**_r_* = 224.26	*D*_x_ = 1.288 Mg m^−^^3^
Orthorhombic, *P**c**a*2_1_	Mo *K*α radiation, λ = 0.71073 Å
Hall symbol: P 2c -2ac	Cell parameters from 1070 reflections
*a* = 10.0299 (5) Å	θ = 2.8–21.3°
*b* = 8.5391 (4) Å	µ = 0.08 mm^−^^1^
*c* = 27.000 (1) Å	*T* = 120 K
*V* = 2312.5 (2) Å^3^	Prism, colorless
*Z* = 8	0.40 × 0.10 × 0.03 mm

### Data collection

**Table:**

Bruker SMART APEX CCD diffractometer	1783 reflections with *I* > 2σ(*I*)
Radiation source: fine-focus sealed tube	*R*_int_ = 0.091
graphite	θ_max_ = 27.5°, θ_min_ = 2.4°
ω scans	*h* = −12→12
15022 measured reflections	*k* = −11→10
2699 independent reflections	*l* = −35→34

### Refinement

**Table:**

Refinement on *F*^2^	Primary atom site location: structure-invariant direct methods
Least-squares matrix: full	Secondary atom site location: difference Fourier map
*R*[*F*^2^ > 2σ(*F*^2^)] = 0.046	Hydrogen site location: inferred from neighbouring sites
*wR*(*F*^2^) = 0.111	H atoms treated by a mixture of independent and constrained refinement
*S* = 1.00	*w* = 1/[σ^2^(*F*_o_^2^) + (0.0497*P*)^2^ + 0.1971*P*] where *P* = (*F*_o_^2^ + 2*F*_c_^2^)/3
2699 reflections	(Δ/σ)_max_ = 0.001
313 parameters	Δρ_max_ = 0.19 e Å^−^^3^
3 restraints	Δρ_min_ = −0.23 e Å^−^^3^

### Fractional atomic coordinates and isotropic or equivalent isotropic displacement parameters (Å^2^)

**Table:**

	*x*	*y*	*z*	*U*_iso_*/*U*_eq_	
O1	0.0484 (3)	1.2098 (3)	0.50009 (13)	0.0299 (7)	
O2	0.2955 (3)	0.7163 (3)	0.31456 (13)	0.0325 (7)	
N1	0.0514 (5)	0.9631 (5)	0.5645 (2)	0.0218 (10)	
H1	−0.030 (2)	0.991 (4)	0.557 (2)	0.026*	
N2	0.2729 (4)	0.9563 (5)	0.56067 (17)	0.0248 (9)	
N3	0.2988 (6)	0.4652 (5)	0.2512 (2)	0.0232 (11)	
H3	0.2154 (18)	0.496 (4)	0.254 (2)	0.028*	
N4	0.5228 (4)	0.4592 (5)	0.25497 (16)	0.0228 (9)	
C1	0.0158 (8)	1.3554 (8)	0.4819 (3)	0.0241 (15)	
C2	−0.0889 (9)	1.3564 (10)	0.4484 (3)	0.038 (2)	
H2	−0.1315	1.2614	0.4392	0.046*	
C3	−0.1303 (13)	1.4968 (6)	0.4286 (5)	0.046 (3)	
H3A	−0.2025	1.4980	0.4059	0.055*	
C4	−0.0687 (9)	1.6364 (10)	0.4412 (3)	0.0363 (19)	
H4	−0.0977	1.7326	0.4271	0.044*	
C5	0.0356 (9)	1.6329 (9)	0.4745 (3)	0.036 (2)	
H5	0.0789	1.7276	0.4835	0.043*	
C6	0.0775 (12)	1.4931 (5)	0.4947 (4)	0.031 (3)	
H6	0.1494	1.4920	0.5177	0.037*	
C7	0.1627 (4)	1.1991 (5)	0.53097 (18)	0.0251 (10)	
H7A	0.1569	1.2759	0.5584	0.030*	
H7B	0.2446	1.2206	0.5117	0.030*	
C8	0.1653 (6)	1.0354 (7)	0.5510 (2)	0.0227 (13)	
C9	0.0880 (4)	0.8244 (5)	0.58670 (16)	0.0207 (10)	
C10	0.0147 (4)	0.7057 (5)	0.60944 (17)	0.0241 (9)	
H10	−0.0799	0.7088	0.6108	0.029*	
C11	0.0864 (4)	0.5826 (6)	0.63004 (18)	0.0266 (11)	
H11	0.0403	0.5013	0.6469	0.032*	
C12	0.2252 (4)	0.5768 (5)	0.62635 (17)	0.0241 (10)	
H12	0.2713	0.4901	0.6403	0.029*	
C13	0.2976 (4)	0.6931 (5)	0.60308 (15)	0.0239 (9)	
H13	0.3919	0.6867	0.6003	0.029*	
C14	0.2276 (4)	0.8200 (5)	0.58383 (16)	0.0219 (9)	
C15	0.2627 (8)	0.8623 (8)	0.3334 (3)	0.0249 (15)	
C16	0.1578 (8)	0.8609 (10)	0.3667 (3)	0.0348 (19)	
H16	0.1140	0.7654	0.3744	0.042*	
C17	0.1170 (12)	0.9988 (5)	0.3886 (4)	0.035 (3)	
H17	0.0473	0.9978	0.4124	0.042*	
C18	0.1780 (9)	1.1382 (10)	0.3759 (3)	0.0333 (18)	
H18	0.1481	1.2340	0.3899	0.040*	
C19	0.2824 (9)	1.1375 (9)	0.3427 (3)	0.0334 (19)	
H19	0.3246	1.2337	0.3346	0.040*	
C20	0.3278 (12)	0.9996 (4)	0.3207 (4)	0.030 (3)	
H20	0.4002	0.9999	0.2980	0.036*	
C21	0.4121 (4)	0.7020 (6)	0.28515 (19)	0.0288 (11)	
H21A	0.4927	0.7198	0.3055	0.035*	
H21B	0.4110	0.7797	0.2579	0.035*	
C22	0.4117 (5)	0.5409 (7)	0.2650 (2)	0.0211 (12)	
C23	0.3366 (4)	0.3256 (5)	0.22992 (16)	0.0201 (10)	
C24	0.2635 (4)	0.2077 (5)	0.20763 (16)	0.0241 (9)	
H24	0.1689	0.2108	0.2062	0.029*	
C25	0.3346 (4)	0.0861 (6)	0.18773 (18)	0.0262 (11)	
H25	0.2881	0.0041	0.1714	0.031*	
C26	0.4741 (4)	0.0791 (5)	0.19069 (18)	0.0286 (10)	
H26	0.5197	−0.0077	0.1766	0.034*	
C27	0.5464 (4)	0.1960 (5)	0.21372 (16)	0.0260 (10)	
H27	0.6408	0.1905	0.2162	0.031*	
C28	0.4760 (4)	0.3219 (5)	0.23303 (16)	0.0203 (9)	

### Atomic displacement parameters (Å^2^)

**Table:**

	*U*^11^	*U*^22^	*U*^33^	*U*^12^	*U*^13^	*U*^23^
O1	0.0255 (17)	0.0259 (16)	0.0382 (17)	0.0004 (14)	−0.0086 (14)	0.0034 (14)
O2	0.0274 (17)	0.0236 (16)	0.0465 (18)	−0.0025 (13)	0.0146 (15)	−0.0054 (14)
N1	0.007 (2)	0.0248 (18)	0.033 (3)	0.005 (2)	−0.0012 (19)	−0.001 (3)
N2	0.017 (2)	0.0212 (18)	0.037 (2)	0.000 (2)	0.002 (2)	−0.002 (2)
N3	0.015 (3)	0.0226 (18)	0.032 (3)	−0.001 (2)	0.002 (2)	−0.001 (3)
N4	0.014 (2)	0.0246 (19)	0.029 (2)	0.004 (2)	0.0014 (18)	−0.006 (2)
C1	0.023 (3)	0.021 (3)	0.027 (3)	0.006 (2)	0.003 (2)	0.004 (2)
C2	0.029 (4)	0.033 (4)	0.052 (4)	0.002 (3)	−0.006 (3)	0.011 (3)
C3	0.026 (7)	0.053 (8)	0.059 (8)	0.005 (2)	−0.011 (6)	0.015 (3)
C4	0.038 (4)	0.033 (4)	0.039 (3)	0.010 (3)	0.011 (3)	0.013 (3)
C5	0.045 (5)	0.029 (4)	0.032 (3)	−0.002 (3)	−0.002 (3)	0.006 (3)
C6	0.042 (7)	0.034 (5)	0.016 (4)	0.001 (2)	−0.010 (4)	0.0036 (17)
C7	0.016 (2)	0.024 (3)	0.036 (3)	−0.0022 (16)	−0.0023 (17)	0.004 (2)
C8	0.021 (3)	0.021 (2)	0.026 (3)	−0.006 (2)	0.000 (2)	0.003 (3)
C9	0.015 (2)	0.023 (2)	0.024 (2)	−0.0020 (17)	−0.0003 (17)	−0.0032 (19)
C10	0.016 (2)	0.025 (2)	0.031 (2)	−0.0027 (18)	0.0027 (18)	−0.001 (2)
C11	0.032 (3)	0.022 (3)	0.026 (2)	−0.0074 (19)	0.0008 (19)	−0.002 (2)
C12	0.025 (3)	0.018 (2)	0.030 (2)	0.0031 (19)	−0.0003 (19)	−0.0006 (19)
C13	0.016 (2)	0.026 (2)	0.029 (2)	0.0034 (17)	0.0003 (18)	0.0000 (18)
C14	0.017 (2)	0.023 (2)	0.025 (2)	0.0029 (18)	0.0011 (17)	−0.0022 (17)
C15	0.025 (3)	0.024 (3)	0.025 (3)	0.000 (2)	0.000 (2)	−0.004 (2)
C16	0.028 (4)	0.036 (4)	0.040 (4)	−0.007 (3)	0.012 (3)	−0.010 (3)
C17	0.029 (6)	0.040 (6)	0.037 (6)	−0.0004 (19)	0.003 (5)	−0.012 (2)
C18	0.033 (4)	0.035 (4)	0.032 (3)	0.009 (3)	−0.002 (3)	−0.012 (3)
C19	0.044 (4)	0.025 (3)	0.031 (3)	0.001 (3)	−0.004 (3)	0.002 (3)
C20	0.029 (6)	0.023 (5)	0.038 (5)	0.0021 (17)	−0.006 (4)	−0.0012 (18)
C21	0.018 (3)	0.033 (3)	0.036 (3)	−0.0038 (18)	0.0055 (18)	−0.004 (2)
C22	0.010 (3)	0.025 (2)	0.029 (3)	0.002 (2)	0.0020 (19)	0.005 (3)
C23	0.019 (2)	0.020 (2)	0.021 (2)	0.0017 (17)	0.0031 (17)	0.0017 (19)
C24	0.019 (2)	0.026 (2)	0.028 (2)	−0.0038 (18)	−0.0001 (17)	0.003 (2)
C25	0.028 (3)	0.025 (3)	0.026 (2)	−0.0021 (19)	−0.0023 (19)	−0.005 (2)
C26	0.029 (3)	0.027 (3)	0.030 (2)	0.004 (2)	0.001 (2)	−0.003 (2)
C27	0.019 (2)	0.028 (2)	0.032 (2)	0.0029 (18)	0.0016 (18)	0.0038 (19)
C28	0.014 (2)	0.020 (2)	0.026 (2)	−0.0038 (17)	0.0009 (17)	0.0047 (17)

### Geometric parameters (Å, °)

**Table:**

O1—C1	1.376 (8)	C10—H10	0.9500
O1—C7	1.421 (5)	C11—C12	1.397 (6)
O2—C15	1.386 (8)	C11—H11	0.9500
O2—C21	1.419 (5)	C12—C13	1.381 (6)
N1—C8	1.349 (8)	C12—H12	0.9500
N1—C9	1.377 (6)	C13—C14	1.392 (5)
N1—H1	0.880 (10)	C13—H13	0.9500
N2—C8	1.299 (7)	C15—C16	1.383 (11)
N2—C14	1.397 (6)	C15—C20	1.385 (11)
N3—C22	1.357 (8)	C16—C17	1.380 (11)
N3—C23	1.376 (6)	C16—H16	0.9500
N3—H3	0.881 (10)	C17—C18	1.382 (11)
N4—C22	1.342 (7)	C17—H17	0.9500
N4—C28	1.395 (6)	C18—C19	1.377 (13)
C1—C6	1.373 (10)	C18—H18	0.9500
C1—C2	1.386 (12)	C19—C20	1.396 (11)
C2—C3	1.377 (11)	C19—H19	0.9500
C2—H2	0.9500	C20—H20	0.9500
C3—C4	1.384 (12)	C21—C22	1.480 (8)
C3—H3A	0.9500	C21—H21A	0.9900
C4—C5	1.381 (13)	C21—H21B	0.9900
C4—H4	0.9500	C23—C24	1.383 (6)
C5—C6	1.379 (10)	C23—C28	1.402 (5)
C5—H5	0.9500	C24—C25	1.369 (6)
C6—H6	0.9500	C24—H24	0.9500
C7—C8	1.500 (7)	C25—C26	1.402 (6)
C7—H7A	0.9900	C25—H25	0.9500
C7—H7B	0.9900	C26—C27	1.382 (6)
C9—C10	1.394 (6)	C26—H26	0.9500
C9—C14	1.403 (5)	C27—C28	1.387 (6)
C10—C11	1.389 (6)	C27—H27	0.9500
			
C1—O1—C7	117.3 (4)	C12—C13—H13	121.2
C15—O2—C21	118.5 (4)	C14—C13—H13	121.2
C8—N1—C9	106.6 (5)	C13—C14—N2	130.7 (4)
C8—N1—H1	127 (3)	C13—C14—C9	120.2 (4)
C9—N1—H1	126 (3)	N2—C14—C9	109.1 (3)
C8—N2—C14	104.6 (5)	C16—C15—C20	121.8 (8)
C22—N3—C23	107.3 (5)	C16—C15—O2	114.3 (6)
C22—N3—H3	129 (3)	C20—C15—O2	124.0 (7)
C23—N3—H3	124 (3)	C17—C16—C15	119.8 (8)
C22—N4—C28	104.1 (4)	C17—C16—H16	120.1
C6—C1—O1	125.2 (7)	C15—C16—H16	120.1
C6—C1—C2	120.1 (8)	C16—C17—C18	119.7 (10)
O1—C1—C2	114.7 (7)	C16—C17—H17	120.1
C3—C2—C1	119.1 (9)	C18—C17—H17	120.1
C3—C2—H2	120.4	C19—C18—C17	119.7 (8)
C1—C2—H2	120.4	C19—C18—H18	120.2
C2—C3—C4	121.4 (11)	C17—C18—H18	120.2
C2—C3—H3A	119.3	C18—C19—C20	121.9 (9)
C4—C3—H3A	119.3	C18—C19—H19	119.0
C5—C4—C3	118.6 (8)	C20—C19—H19	119.0
C5—C4—H4	120.7	C15—C20—C19	117.0 (11)
C3—C4—H4	120.7	C15—C20—H20	121.5
C6—C5—C4	120.6 (9)	C19—C20—H20	121.5
C6—C5—H5	119.7	O2—C21—C22	106.5 (4)
C4—C5—H5	119.7	O2—C21—H21A	110.4
C1—C6—C5	120.2 (10)	C22—C21—H21A	110.4
C1—C6—H6	119.9	O2—C21—H21B	110.4
C5—C6—H6	119.9	C22—C21—H21B	110.4
O1—C7—C8	106.6 (4)	H21A—C21—H21B	108.6
O1—C7—H7A	110.4	N4—C22—N3	113.0 (5)
C8—C7—H7A	110.4	N4—C22—C21	123.7 (5)
O1—C7—H7B	110.4	N3—C22—C21	123.1 (5)
C8—C7—H7B	110.4	N3—C23—C24	131.8 (4)
H7A—C7—H7B	108.6	N3—C23—C28	105.7 (4)
N2—C8—N1	114.3 (5)	C24—C23—C28	122.5 (4)
N2—C8—C7	124.8 (5)	C25—C24—C23	116.6 (4)
N1—C8—C7	120.6 (5)	C25—C24—H24	121.7
N1—C9—C10	132.6 (4)	C23—C24—H24	121.7
N1—C9—C14	105.3 (4)	C24—C25—C26	122.0 (4)
C10—C9—C14	122.1 (4)	C24—C25—H25	119.0
C11—C10—C9	117.0 (4)	C26—C25—H25	119.0
C11—C10—H10	121.5	C27—C26—C25	121.2 (4)
C9—C10—H10	121.5	C27—C26—H26	119.4
C10—C11—C12	120.9 (4)	C25—C26—H26	119.4
C10—C11—H11	119.5	C26—C27—C28	117.5 (4)
C12—C11—H11	119.5	C26—C27—H27	121.3
C13—C12—C11	122.0 (4)	C28—C27—H27	121.3
C13—C12—H12	119.0	C27—C28—N4	129.8 (4)
C11—C12—H12	119.0	C27—C28—C23	120.2 (4)
C12—C13—C14	117.7 (4)	N4—C28—C23	110.0 (3)
			
C7—O1—C1—C6	−6.1 (11)	C21—O2—C15—C16	−172.0 (6)
C7—O1—C1—C2	174.2 (6)	C21—O2—C15—C20	7.9 (10)
C6—C1—C2—C3	−0.5 (15)	C20—C15—C16—C17	−0.9 (14)
O1—C1—C2—C3	179.1 (8)	O2—C15—C16—C17	179.0 (8)
C1—C2—C3—C4	0.6 (18)	C15—C16—C17—C18	2.4 (16)
C2—C3—C4—C5	−0.4 (18)	C16—C17—C18—C19	−2.4 (16)
C3—C4—C5—C6	0.0 (15)	C17—C18—C19—C20	1.1 (14)
O1—C1—C6—C5	−179.4 (6)	C16—C15—C20—C19	−0.4 (15)
C2—C1—C6—C5	0.2 (16)	O2—C15—C20—C19	179.6 (6)
C4—C5—C6—C1	0.1 (16)	C18—C19—C20—C15	0.4 (15)
C1—O1—C7—C8	172.5 (5)	C15—O2—C21—C22	−172.6 (5)
C14—N2—C8—N1	−1.5 (7)	C28—N4—C22—N3	0.2 (7)
C14—N2—C8—C7	173.1 (5)	C28—N4—C22—C21	−175.0 (5)
C9—N1—C8—N2	2.0 (8)	C23—N3—C22—N4	−1.0 (7)
C9—N1—C8—C7	−172.9 (5)	C23—N3—C22—C21	174.3 (5)
O1—C7—C8—N2	146.8 (6)	O2—C21—C22—N4	−149.1 (5)
O1—C7—C8—N1	−39.0 (7)	O2—C21—C22—N3	36.1 (7)
C8—N1—C9—C10	177.0 (5)	C22—N3—C23—C24	−176.6 (5)
C8—N1—C9—C14	−1.6 (6)	C22—N3—C23—C28	1.3 (6)
N1—C9—C10—C11	−177.4 (5)	N3—C23—C24—C25	176.7 (5)
C14—C9—C10—C11	1.0 (6)	C28—C23—C24—C25	−1.0 (6)
C9—C10—C11—C12	−2.2 (6)	C23—C24—C25—C26	1.5 (7)
C10—C11—C12—C13	1.2 (6)	C24—C25—C26—C27	−0.5 (7)
C11—C12—C13—C14	1.1 (6)	C25—C26—C27—C28	−1.1 (6)
C12—C13—C14—N2	176.8 (4)	C26—C27—C28—N4	−175.4 (4)
C12—C13—C14—C9	−2.3 (5)	C26—C27—C28—C23	1.5 (6)
C8—N2—C14—C13	−178.8 (5)	C22—N4—C28—C27	177.8 (5)
C8—N2—C14—C9	0.4 (6)	C22—N4—C28—C23	0.7 (5)
N1—C9—C14—C13	−179.9 (4)	N3—C23—C28—C27	−178.7 (4)
C10—C9—C14—C13	1.3 (6)	C24—C23—C28—C27	−0.5 (6)
N1—C9—C14—N2	0.7 (5)	N3—C23—C28—N4	−1.3 (5)
C10—C9—C14—N2	−178.0 (4)	C24—C23—C28—N4	176.9 (4)

### Hydrogen-bond geometry (Å, °)

**Table:**

*D*—H···*A*	*D*—H	H···*A*	*D*···*A*	*D*—H···*A*
N1—H1···N2^i^	0.88 (1)	2.03 (2)	2.879 (7)	163 (6)
N3—H3···N4^ii^	0.88 (1)	1.97 (2)	2.845 (8)	172 (5)

Symmetry codes: (i) *x*−1/2, −*y*+2, *z*; (ii) *x*−1/2, −*y*+1, *z*.
